# The Role of Raf Kinase Inhibitor Protein (RKIP) in HER2+ Breast Cancer Immune Evasion

**DOI:** 10.3390/cells15040319

**Published:** 2026-02-08

**Authors:** Ania Khachikian, Mai Ho, Benjamin Bonavida

**Affiliations:** Department of Microbiology, Immunology & Molecular Genetics, David Geffen School of Medicine, Jonsson Comprehensive Cancer Center, University of California, Los Angeles, CA 90095, USA; khachikianania@gmail.com (A.K.); maiho123@ucla.edu (M.H.)

**Keywords:** breast cancer, RKIP, HER2, immune evasion, cross-talk signaling, targeting, immunotherapy

## Abstract

Breast cancer (BC) is a prevalent malignancy worldwide among women. HER2 overexpression in a subset of BC (HER2+ BC) serves as a critical oncogenic driver and contributes to immune evasion. The Raf Kinase Inhibitor Protein (RKIP), a metastasis suppressor and an immune enhancer, is underexpressed in HER2+ BC. The treatment of HER2+ BC with anti-HER2 mAbs or chemical inhibitors has resulted in significant clinical responses in a subset of patients; however, unresponsiveness in a larger subset was due to acquired and induced resistance. These findings highlight the need for the development of new effective therapies. By analyzing the signaling pathways mediated by both RKIP and HER2 in HER2+ BC, we have found that RKIP and HER2 downstream signaling and inductions showed an inverse relationship. These suggested the presence of a dysregulated RKIP-HER2 axis in HER2+ BC mediating immune evasion. These findings were corroborated by bioinformatic analyses. The immune evasion induced by the overexpression of HER2 was due, in part, to its regulation of the expression of PD-L1, the polarization of TAMs, the infiltration of suppressor cells (Tregs, MDSCs), and the inhibition of anti-tumor CD8+ T cells, resulting in an overall immunosuppressive TME. In contrast, RKIP expression inhibits critical signaling pathways that regulate HER2 expression, including the Raf-MEK-ERK, NF-kB, and PI3K/Akt pathways, thereby aborting HER2-mediated mechanisms of immune evasion. Overall, we analyzed the cross-talk signaling pathways between RKIP and HER2, established a novel dysregulated axis in HER2+ BC, and delineated the various mechanisms involved in the regulation of immune evasion by RKIP and HER2. Hence, we present various therapeutic strategies aimed at targeting the RKIP-HER2 axis in HER2+ BC to circumvent unresponsiveness to therapeutics and immune evasion.

## 1. Introduction

Breast cancer (BC) is a leading malignancy affecting millions globally, predominantly women, with 2.3 million new cases and 670,000 deaths reported in 2022 [1]. If current trends continue, the concerning rise in incidence and mortality rates will reach 38% and 68%, respectively, by 2050 [2]. BC is highly heterogeneous, further classified into molecular subtypes like luminal A, luminal B, human epidermal receptor 2 (HER2)-positive BC, and triple-negative BC (TNBC) [3].

HER2+ BC accounts for approximately 15–20% of all BC diagnoses [4]. Historically, the HER2 protein has been considered one of the most aggressive markers in BC and is strongly associated with poor prognosis [5]. However, the development of trastuzumab (Herceptin), the first anti-HER2 agent, has led to significant improvements in survival outcomes for a subset of patients with HER2+ BC—especially when treated at earlier stages [6]. Subsequent monoclonal antibodies (mAbs), including pertuzumab and margetuximab, along with small-molecule inhibitors like lapatinib, similarly target HER1/HER2 kinases to block the formation of HER2–HER2 homodimers and ligand-independent HER2–HER3, HER2–HER1, and HER2–HER4 heterodimers [7]. Newer versions of trastuzumab, such as T-DXd (trastuzumab deruxtecan), can act as an antibody-drug conjugate (ADC) to deliver a potent chemotherapy payload directly to the cancer cell, reducing systemic toxicity [8,9].

While the above treatment modalities have significantly improved survival rates for patients with HER2+ BC, the development of metastatic HER2+ tumors will eventually lead to acquired or de novo resistance in the majority of patients [3]. In cases where HER2+ BC tumors eventually recur, the continued reliance of tumors on HER2 signaling underscores the need for novel therapeutic strategies. Ongoing research across preclinical and clinical domains aims to develop the next-generation HER2-targeted treatments that can overcome resistance and further improve outcomes for patients with HER2+ BC. Such undertakings also seek to target factors beyond HER2, designing novel combination strategies that can simultaneously target proteins such as Programmed Death-Ligand 1 (PD-L1), Cytotoxic T-Lymphocyte-Associated Protein 4 (CTLA4), Natural Killer Group 2 family of receptor A (NKG2A), A Serine/Threonine Protein Kinase (Akt), and phosphoinositide 3-kinase (PI3K) and provide a molecular basis to explore the HER2′s regulatory network with other gene products in the tumor microenvironment (TME), including key interactions with proteins such as the Raf Kinase Inhibitory Protein (RKIP) [10,11].

RKIP has emerged as a promising therapeutic target for various solid cancers, including BC. Initially identified as part of the phosphatidylcholine-binding protein (PEBP) superfamily, RKIP is known for inhibiting the Raf-MEK-ERK signaling pathway. By preventing MEK phosphorylation, RKIP blocks downstream activation of extracellular signal-regulated kinase (ERK), thereby suppressing the oncogenic Raf-MEK-ERK (MAPK) signaling pathway, which is upregulated in almost 40% of all cancers [12,13,14]. Elevated RKIP levels are often associated with improved clinical outcomes, and their overexpression reverses resistance to conventional therapies [15]. Conversely, reduced RKIP expression is associated with increased metastasis, tumor aggressiveness, and chemoresistance [16,17,18,19]. RKIP regulates the infiltration of specific immune cells and the secretion of anti-metastatic factors that are critical to address immune evasion mechanisms present in recurrent HER2+ BC [20,21].

The main objective of this review is to examine the regulation of RKIP and HER2 expressions in HER2+ BC, analyze the cross-talk signaling pathways between RKIP and HER2, establish a novel dysregulated RKIP-HER2 axis in HER2+ BC, delineate the underlying mechanisms involved in immune evasion by RKIP and HER2, explore the axis as a potential therapeutic target, address challenges associated with RKIP and HER2 targeting agents directly on the cancer cells, and offer future perspectives.

## 2. RKIP and HER2 Expressions in HER2+ BC

### 2.1. RKIP

Different subtypes of BC exhibit varying levels of RKIP expression. RKIP is significantly downregulated in HER2+, luminal, and TNBC tissues compared with normal breast tissue, as derived from analyses using the UALCAN cancer-omics portal, which integrates public datasets from The Cancer Genome Atlas (TCGA) and the Clinical Proteomic Tumor Analysis Consortium (CPTAC). UALCAN allows users to query RKIP in BC and visualize expression across normal tissue and molecular subtypes (including HER2-enriched, luminal, and basal/TNBC), from which we observed lower RKIP levels in these tumor subtypes relative to normal breast samples. RKIP expression varies significantly across BC subtypes. Luminal A and B tumors generally exhibit higher RKIP expressions, whereas HER2-enriched, basal-like, and claudin-low subtypes show reduced RKIP levels. In HER2+ BC specifically, RKIP is consistently downregulated at the protein level compared to normal breast tissue and luminal tumors, despite variable mRNA expression [3,22,23]. This reduction aligns with aggressive tumor behavior, enhanced HER2 signaling, immune evasion, and therapy resistance. These findings are summarized in a dedicated table focusing on HER2+ BC (Table 1).

In various aggressive BC subtypes, RKIP expression may be lost or significantly reduced [15]. In the study by Lai et al. [15], two types of BC cells were analyzed to establish RKIP as a promising metastasis suppressor. MCF-7 cells with high RKIP expression levels and MDA-MB-231 cells with low RKIP levels were used. Manipulating RKIP in different experimental settings revealed an uneven migration between MCF-7 and MDA-MB-231 cells, which was mainly characterized by differences in RKIP expression levels [15]. In BC, lower RKIP levels have been correlated with specific tumor sizes and grades [23]. Additionally, RKIP expression levels are known to be drastically induced during chemotherapeutic drug treatment, with the highest levels of RKIP correlating with higher apoptosis rates [24]. Briefly, Li et al. demonstrated that RKIP expression is inversely associated with the invasiveness of MDA-MB-435 cells [25]. In addition, Zhang et al. reported a significant negative correlation between RKIP expression and HER-2/neu status (*p* = 0.013), deeming RKIP as a negative predictor of the HER2/neu status [26]. In vitro, they further demonstrated a dose-dependent reduction in RKIP expression in BT-474 cells upon HER2/neu modulation. We have also expanded on the discussion of the study by Al-Mulla et al., whereby they demonstrated that HER2-enriched breast tumors ranked second lowest in terms of RKIP expression using three independent BC cohorts [23].

### 2.2. HER2

HER2 is a member of the epidermal growth factor receptor (EGFR) family. It is a transmembrane glycoprotein that plays a significant role in influencing cell proliferation and differentiation [11]. Patients diagnosed with HER2+ BC are not always fully cured, and those who acquire the HER2+ metastatic variant cultivate a very deadly disease [27,28]. HER2 overexpression in BC accounts for approximately 15–20% of all cases and is characterized by HER2 gene amplification or HER2 protein overexpression [28].

Unlike most receptors, HER2 does not require a ligand for activation; instead, it becomes active by forming dimers [11]. The key signaling pathways involving HER2 include PI3K/Akt, MAPK, and NF-κB. The PI3K pathway involves Akt phosphorylation, which activates the downstream mammalian target of rapamycin (mTOR) through a negative feedback loop while simultaneously inhibiting the mammalian target of rapamycin complex 1 (mTORC1) [11,29,30]. This interaction activates PI3K, converting PIP2 to PIP3, which recruits Akt and triggers downstream signaling [31]. HER2′s stronger, sustained signaling enhances Akt activation and mTOR activity, promoting cell survival, growth, and resistance to apoptosis [32]. Thus, HER2 overexpression amplifies PI3K signaling, contributing to oncogenic transformation and therapeutic resistance.

## 3. Regulation of RKIP and HER2 Expressions in HER2+ BC

Regulation at the transcriptional, epigenetic, and post-translational levels is crucial for understanding RKIP and HER2 regulation in HER2+ BC. RKIP expression levels and molecular assemblies with various protein partners are essential for regulating the progression and metastatic potential of HER2+ BC cells. As such, the absence of RKIP allows multiple oncogenic genes, such as HER2, to be expressed, contributing to cancer progression [33,34]. Below, we summarize key modulators of RKIP and HER2 expressions, transcriptionally and post-transcriptionally.

### 3.1. RKIP

Various transcription factors (TFs) regulate RKIP expression [35]. A significant RKIP regulator is SNAIL, an EMT protein that can transcriptionally repress RKIP [16,35]. The induction of SNAIL can be promoted through NFκB and its downstream target Yin Yang 1 (YY1), thereby repressing RKIP feedback [16,36,37]. The overexpression of SNAIL and YY1 is common in many cancers, while RKIP is downregulated [38]. Another significant factor in cancer metastasis is the basic leucine zipper transcription factor 1 (BACH1) [18,35,36,37,38]. RKIP and BACH1 work together by regulating each other; an anti-metastatic state corresponds with high RKIP and low BACH1 expressions, while a pro-metastatic state corresponds with RKIP inhibition [39]. In addition, it is essential to highlight that RKIP inhibits BACH1 by targeting the Erk1/2-myc-let7 pathway [40]. Aside from SNAIL and BACH1, RKIP transcription is modulated by the Enhancer of Zeste Homolog 2 (EZH2), an enzymatic subunit of the Polycomb Repressive Complex 2 (PRC2) [41,42]. At the epigenetic level, histone deacetylation and DNA methylation can tighten chromatin structure at the promoters of the regulator of G protein signaling (RGS) genes, obstructing transcriptional access [41]. At this level, the RKIP promoter is methylated in most cancers [41]. As such, both SNAIL and YY1 can repress RKIP transcription by recruiting the EZH2 repressive complex to the proximal RKIP promoter [41] (Table 2).

Regarding post-transcriptional regulation, miRNAs play a significant role in targeting RKIP mRNA for degradation. miRNA complexes can bind to miRNA recognition elements (MREs) on target mRNA within the 3′ untranslated region (3′UTR). This binding leads to silencing of mRNA through cleavage, destabilization, or reduced translation efficiency [43]. Several miRNAs target RKIP mRNA and inhibit its expression, a phenomenon observed across various cancer types. For instance, in prostate cancer, miR-543 and miR-23a have been identified as suppressors of RKIP expression [44,45]. In lung cancer, miR-27a is an RKIP suppressor [45], and in BC, miR-224 has been reported to suppress RKIP expression [46]. MiR-224, miR-27a, miR-23a, and miR-543 target and inhibit RKIP transcription [34,44,45,46,47,48] (Table 2).

At the protein level, post-translational modifications can affect RKIP stability and function [49]. Studies by Skinner et al. [49] demonstrate that RKIP phosphorylation controls the stability of RKIP kinase, thereby affecting its state and ability to regulate. Phosphorylation of RKIP at Serine 153 (pSer153-RKIP) by protein kinase C ζ (PKCζ) redirects RKIP to bind and inhibit GRK2 instead of Raf-1, thereby functioning as a proto-oncogene [50,51,52,53,54,55,56]. However, there have not been any reports specifically for HER2+BC, and that needs to be further investigated. Phosphorylation of RKIP at specific residues (notably Ser153 by PKCζ) alters RKIP’s binding partners and functional role. Instead of inhibiting Raf-1, phosphorylated RKIP preferentially inhibits GRK2, which can promote GPCR signaling, cell migration, invasion, and therapy resistance. This functional switch has been shown to support oncogenic behavior in several cancers. As such, in certain cancers, pSer153-RKIP is associated with poor therapy response and prognosis while also amplifying the proliferation and invasion of cancerous cells by inhibiting GRK2 [13,54,57]. Moreover, RKIP exists in multiple states and features an allosteric structure controlled by phosphorylation and dynamics within a pocket loop [49,58]. With that said, the impact of RKIP phosphorylation extends to promoting signaling degradation, autophagy via LC3 and Rab8, invasion via IQGAP proteins, and mechanosensing [59] (Table 2). The regulation of RKIP is summarized in Table 2.

**Table 2:** Regulation of RKIP expression.

Types of Regulation	Factors Involved	Effect on RKIP	References
Transcriptional	- SNAIL, NFκB, YY1 - BACH1 - EZH2 + PRC2	- Downregulation of RKIP - RKIP and BACH1 regulate each other and foster a negatively correlated relationship - EZH2 and PRC2 are repressive complexes	[16,18,35,36,37,38,39,41,42]
Epigenetic	- RKIP promoter methylation	- Leads to RKIP silencing (reduction in RKIP expression when the promoter undergoes methylation)	[44,60,61,62]
Post-Transcriptional	- MicroRNAs and mRNA-binding proteins - PKCζ phosphorylation	- Many miRNAs inhibit RKIP expression (e.g., miR-224) - Suppresses RKIP activity (Ser153-RKIP by PKCζ leads to RKIP activity loss)	[48,50,51,52,53,54,55,56,63,64]
Translational	- Global and mRNA-specific mechanisms	- RKIP is able to regulate processes crucial for tumor growth and treatment resistance	[65,66]

This table illustrates the key factors that regulate RKIP expression. RKIP is pivotal in regulating key signaling pathways and gene expression, significantly impacting cancer progression, metastasis, and therapeutic resistance.

### 3.2. HER2

At the transcriptional level, several TFs influence the upregulation or downregulation of HER2. HER2 also translocates into the nucleus, where it is involved in transcriptional activity, associating with p185neu to activate transcription [67,68,69,70]. TFs associated with HER2 include the transcription factor family activator protein 2 (TFAP2) [71,72,73], the specificity protein 1 (Sp1) [74], the palindrome binding protein (PBP) [75], the YY1 [76], the E26 transformation specific (ETS) [77], the Y-box binding protein-1 (YB-1) [78,79], the early growth response protein 2 (EGR2) [80], the myeloblastosis (MYB) [81], the forkhead box protein P3 (FOXP3) [82], the GATA-binding protein 4 (GATA4) [83], the polyomavirus enhancer activator 3 (PEA3) [84], the c-MYC promoter-binding protein-1 (MBP-1) [85,86], the membrane bound NOTCH and the recombination signal binding protein for immunoglobulin kappa J region (RBP-Jk) [87,88]. Of those TFs listed, seven are known to upregulate HER2: TFAP2, Sp1, PBP, YY1, ETS, YB-1, and EGR2 [89]. For example, a report by Begon et al. [76] examined YY1 and its interactions with the activator protein 2 (AP-2), supporting the notion that YY1 and AP-2 collaborate to promote the HER2 oncogene by binding to AP-2 sites [76]. However, the remaining TFs—MYB, FOXP3, GATA4, PEA3, MBP-1, NOTCH, and RBP-Jk—have been shown to negatively regulate HER2 expression in BC [89]. More specifically, MYB can repress HER2 despite its role as a transcriptional activator [81]. FOXP3 binds to the HER2/ErbB2 promoter and suppresses its transcription. In addition, an inverse correlation between FOXP3 and HER2 levels has also been observed in tumor samples [82]. GATA4 has been detected in breast tumors and contributes to the transcriptional control of HER2. Additionally, MBP-1, NOTCH, and RBP-Jk are associated with ERbB2 but also are significant in negatively influencing HER2 [86,87]. At the transcriptional level, adenovirus early region 1A (E1A) is responsible for repressing HER2 by inactivating and inhibiting the p300/CBP complex [87,90,91,92,93,94] (Table 3).

Epigenetic and genetic mechanisms work hand in hand to control cancer proliferation [95]. DNA methylation, histone modifications, non-coding RNAs (ncRNAs), and miRNAs collectively constitute epigenetic mechanisms that contribute to cancer heterogeneity [96]. For example, histone modifications, such as dysregulation, are linked to the HER2 chromatin complex, while ncRNAs aim to weaken proteins essential to the HER2 signaling pathway [95,97]. Histone modifications in the HER2 gene body enhancer (HGE) from intron 19 to intron 22 can promote transcriptional activity [89]. However, in terms of DNA methylation, HER2 promoter methylation results in HER2 downregulation, whereas demethylation results in HER2 upregulation [94,98]. On the same note, DNA methylation in the HGE region shows an inverse relationship with HER2 expression and can inhibit histone modifications that could enhance transcription of HER2 promoters [89]. In a study by Liu et al. [47], histone three lysine four trimethylation (H3K4me3) and histone three lysine nine acetylation (H3K9ac) are critical in active transcription and co-localize at the HER2 promoter region [89,99,100,101,102]. The HGE was a significant finding, enhancing transcription at HER2 promoters through its histone modification marks [89]. This was demonstrated using a luciferase reporter assay, incorporating 293T, SKBR3, and BT474 cell lines. The study also highlighted that TFAP2C positively influences HGE. Therefore, epigenetic factors can also affect accessibility to transcriptional mechanisms [11,103] (Table 3).

Regarding post-transcriptional regulation, lncRNAs are paramount in gene expression regulation [131,132]. Compared to other types of BCs involving HER2, such as HER2+/ER+/PR+ and HER2-negative/ER+/PR+ BCs, LINK-A expression levels were higher in TNBC, particularly in stage III [104].

Alongside lncRNAs, multiple microRNAs (miRNAs) play critical roles in HER2 post-transcriptional regulation. Specific miRNAs such as miR-342-5p, miR-124, and miR-193a-3p have been shown to directly target ERBB2 mRNA, which encodes the HER2 protein, by binding to its 3′ untranslated region and suppressing its translation.

Notably, miRNAs are relevant in clinical settings, as they can serve as a prognostic signature, enabling patients to receive personalized treatment based on their unique responses [105,106]. Although not all of the miRNAs discussed directly affect ERBB2 mRNA, many of them—such as miR-96, miR-10b, miR-17, miR-148a, and miR-335-5p—modulate HER2-related signaling pathways, thereby impacting proliferation, apoptosis, and metastasis in HER2+ BC [107] (Table 3).

An essential aspect of HER2 regulation involves translational control and protein stability. Translation may be inhibited as miRNAs bind to the 3′UTR of targeted mRNAs to regulate HER2 expression post-transcriptionally [11]. However, in terms of stability, various regulatory proteins intervene in dimerization. For instance, Hsp90 is critical in cellular signaling and cell survival [120]. As a chaperone protein, Hsp90 can stabilize HER2 and act as a therapeutic target in many disorders, infections, and cancers [11,120,121,122]. In addition, specific regulatory proteins like Hsp90 are also involved in HER2 activity, which helps regulate tyrosine kinase activity and dimerization [11]. The proteasome plays a key role in this process, mediating the degradation of cellular proteins [123,124]. Proteasomes are crucial in DNA repair and cell proliferation in both normal and malignant cells [125,126]. Similarly, proteasome inhibitors (PIs) are also involved in cell survival and anti-cancer activity [126]. In cancerous cells, PIs induce apoptosis through various mechanisms, such as NF-kB inhibition [126]. The NF-kB pathway is deactivated when IκBα remains intact and bound to the heterodimer p50/p65, as proteasome activity is suppressed by inhibition [126]. PIs are relevant to HER2 in BC, as they may have therapeutic relevance [127]. PIs may suppress mutant HER2 activity while reversing autophosphorylation [127]. Furthermore, a study by Thaler et al. [127] suggests that PIs may also target HER2-negative/ER+ BC and serve as a therapeutic regimen [127,128].

Regarding HER2 stability, protein tyrosine phosphatase non-receptor type 18 (PTPN18) has been shown to function as a negative regulator [108,129]. The active site of PTPN18, where catalytic reactions occur, mediates dephosphorylation of HER2 at the pY site, Y1112, which interferes with c-Cbl recruitment and delays HER2 degradation by limiting lysosomal trafficking [108,130]. In BC, elevated expression of PTPN18 has been correlated with HER2 protein levels, suggesting that PTPN18 may contribute to sustained HER2 signaling and tumor progression [108]. Table 3 outlines the key mechanisms regulating HER2 expression.

## 4. The Dysregulated RKIP-HER2 Axis in HER2+ BC

RKIP plays a central role in regulating oncogenic signaling pathways, many of which are often dysregulated in HER2+ BCs. HER2 expression is positively regulated by (i) the MAPK pathway, (ii) the NF-kB pathway, and (iii) the PI3K/Akt pathway, and RKIP negatively regulates each to varying degrees [12,133,134,135]. HER2 and RKIP co-regulate these major signaling pathways in an antagonistic manner, with HER2 acting as an upstream activator and RKIP serving as a suppressor of pathway activities.

The inhibitory function of RKIP has been demonstrated in HER2-overexpressing BT-474 BC cells, where RKIP reintroduction led to decreased ERK activity and impaired cellular proliferation. Similarly, NF-κB signaling, another downstream effector of HER2, is blocked by RKIP through inhibition of upstream kinases such as TAK1 and NIK, preventing NF-κB nuclear translocation and limiting pro-inflammatory transcription [23,24]. HER2 itself can activate NF-κB through PI3K/Akt-mediated phosphorylation of IKKa, promoting the transcription of NF-κB target genes involved in survival, inflammation, and immune evasion [24]. RKIP loss has also been linked to the epithelial–mesenchymal transition (EMT), a critical driver of metastasis and therapeutic resistance in HER2+ BCs. HER2 promotes EMT by activating TFs such as SNAIL and ZEB-1 [135,136,137,138,139,140,141]. In contrast, RKIP suppresses EMT by inhibiting NF-κB, repressing SNAIL transcription, and negatively regulating STAT3 activity, a major TF involved in tumor progression and resistance to HER2-targeted therapies [134,142,143]. This relationship between HER2 signaling and RKIP suggests an intricate axis in which HER2 promotes EMT and metastasis, while RKIP acts as a counter-regulatory molecule inhibiting these processes.

**Figure 1:**
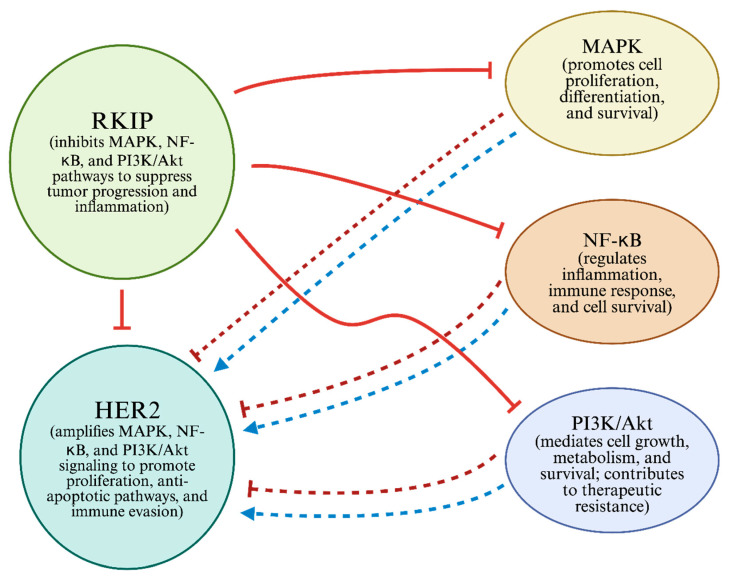
RKIP and HER2 cross-talk signaling. The MAPK, NF-κB, and Akt pathways regulated by HER2 expression are shown (⇢). RKIP blocks critical signaling pathways, such as the MAPK, NF-κB, and PI3K/Akt pathways (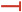) [144,145,146,147]. However, by inhibiting these signaling cascades, RKIP reduces HER2-related signaling [12,133,134,135,136]. This figure demonstrates the antagonistic relationship between RKIP and HER2 and the regulatory feedback loop they form. Additionally, it illustrates three common mechanisms by which RKIP and HER2 interact, influencing cancer treatment and HER2-mediated cell proliferation. Created with BioRender.com. Accessed on 1 November 2025.

The opposing activities of HER2 and RKIP form a regulatory axis that determines the strength and persistence of oncogenic signaling in HER2+ BC. A defining feature of the RKIP-HER2 axis is the inverse correlation between HER2 overexpression and RKIP downregulation. While HER2 promotes oncogenic signaling and immune evasion, RKIP acts as a counterbalance, suppressing MAPK, NF-κB, and PI3K/Akt activities [144] (see Figure 1). In a recent study, Cardoso-Carneiro et al. [144] demonstrated a consistent inverse correlation between RKIP and EGFR expression across 25 of 30 solid tumor types in cervical cancer models. Their analyses revealed a feedback loop where RKIP loss increased EGFR transcription and phosphorylation, while EGFR overactivation suppressed RKIP expression. Although these findings are not BC-specific, the conserved role of HER-family signaling across epithelial tumors suggests that these findings are highly relevant to HER2+ BC. Given that HER2 and EGFR share structural and functional homologies and activate overlapping downstream pathways, the amplification of EGFR signaling upon RKIP loss supports a broader paradigm in which RKIP downregulation may similarly amplify HER2-driven oncogenic and immunosuppressive pathways in BC.

Altogether, the above findings highlight the RKIP–HER2 axis as a complex regulatory network in HER2-driven BCs, characterized by dynamic interplay between RKIP-mediated signaling suppression and HER2-driven oncogenic activation that collectively shapes tumor progression, therapeutic response, and disease aggressiveness. Loss of RKIP permits sustained HER2 signaling, immune suppression, and metastasis, whereas its restoration can simultaneously impair multiple tumor-promoting pathways. A deeper mechanistic understanding of RKIP’s role, especially regarding EMT, STAT3, and immunogenicity, may inform the development of more effective, targeted therapies. RKIP downregulation and HER2 activation reshape the tumor microenvironment and the immune response, underscoring their roles in immune evasion and setting the stage for further exploration of their immunological impact (Figure 1).

## 5. The RKIP-HER2 Axis in HER2+ BC and Immune Evasion

**Figure 2:**
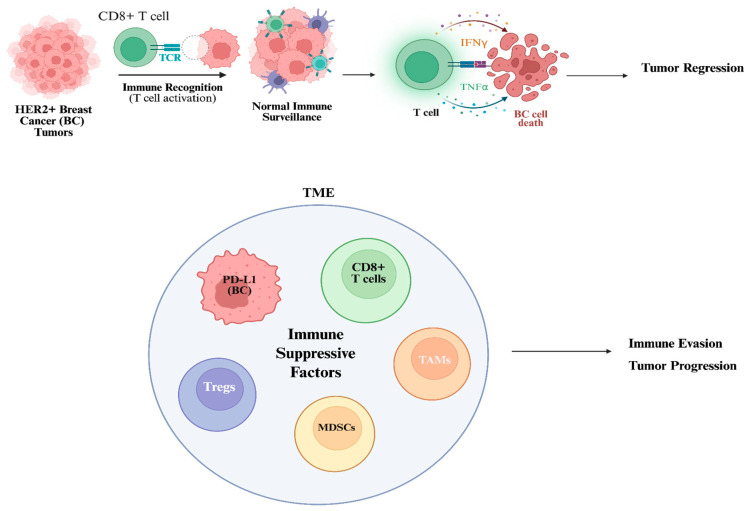
HER2+ BC immune recognition and evasion. The top figure represents immune surveillance via the recognition of the anti-tumor CD8+ T cells targeting the HER2+ BC cells and leading to their cell death. This results in tumor regression. The bottom figure represents the tumor microenvironment (TME) in which multiple cells and factors combined lead to immune evasion. In the TME are represented the tumor cells expressing PD-L1, CD8+ T cells expressing PD1, the infiltration of Tregs, MDSCs, and TAMs cells, and, in addition, various immunosuppressive factors are derived from these cells. This leads to immune evasion and tumor progression. Created with BioRender.com. Accessed on 12 November 2025.

Immune evasion refers to the strategies by which cancer cells escape recognition and destruction by the immune system. These strategies fall into three broad categories—camouflage, coercion, and cytoprotection—as outlined in the conceptual framework proposed by Galassi and colleagues [147]. Within this, it involves antigen presentation, immune checkpoint molecules (e.g., PD-1/PD-L1), tumor plasticity, immunosuppressive immune cells, and T-cell exhaustion (Figure 2).

In HER2+ BC, trastuzumab-resistant tumors can exploit immune escape mechanisms to sustain tumor progression [148]. Such strategies include upregulating immune checkpoint proteins and recruiting immunosuppressive signals [149]. For example, Martinez et al. [149] reported a mechanism where BC cells can overexpress the neuropeptide, Neuromedin U (NmU), and their extracellular vesicles (EVs) to increase levels of transforming growth factor beta-1 (TGFβ1) and PD-L1, contributing to enhanced resistance to antibody-dependent cell cytotoxicity (ADCC) [149]. This mechanism is highly important, given that ADCC is one of trastuzumab’s primary mechanisms of action [149,150]. Clinically, TGFβ1 levels were also significantly higher in EVs isolated from the serum of HER2+ BC patients who did not respond to HER2-targeted treatments compared to those who experienced complete or partial responses [149,151]. Cytokines, such as tumor necrosis factor-alpha (TNFα), can also induce recruitment of immunosuppressive immune cells to promote trastuzumab resistance [152] (Figure 2). TNFα can upregulate the transmembrane glycoprotein MUC4 to shield the HER2 epitope through heavy glycosylation, hindering trastuzumab binding and therapeutic effects [152]. MUC4 expression is linked to an immune desert TME with low tumor-infiltrating lymphocytes (TILs), further contributing to immune evasion and poor response to therapy [152,153].

TME infiltration in HER2+ BC has also been associated with other inhibitory immune cells, including Tregs and MDSCs, correlating with poor survival [154] (Figure 2). In a real-world study involving 124 patients with HER2+ metastatic BC (mBC), Steenbruggen et al. [154] found that Treg infiltration was linked to reduced overall survival (OS) across the entire cohort and in patients who achieved radiological complete remission (rCR). Additionally, other T cell subsets, like CD8+ T cells, were associated with a trend toward decreased OS in patients with primary BC. In a study by Bailur et al. [155], patients who exhibited HER2-reactive CD8+ T cell responses and low levels of MDSCs had a 100% 5-year survival rate, in contrast to a 38% rate in those with elevated MDSCs and absent CD8+ responses [155] (Figure 2). Mechanistically, MDSCs may promote tumor progression through the induction of Tregs, as supported by earlier studies [156,157]. Furthermore, Treg infiltration has been linked to activation of the PI3K signaling pathway—a known driver of resistance in HER2+ and ER+ BC—highlighting a potential rationale for combining HER2-targeted therapies with PI3K inhibitors and agents that deplete Tregs [158,159].

In early-stage HER2+ BC, higher densities of stromal CD8+ and FOXP3+ T cells, as well as immune cell aggregates, were associated with achieving pathologic complete response (pCR) to neoadjuvant therapy [160]. Comparative studies indicate that HER2+ tumors harbor significantly higher densities of CD3+, CD8+, and Treg cells than HER2-negative tumors, suggesting a unique immune microenvironment that may influence treatment response [160,161,162,163]. Moreover, molecular subtyping has revealed at least five distinct HER2+ BC subtypes characterized by differences in both tumor-intrinsic and microenvironmental factors, which have allowed for the characterization and validation of molecular subtypes associated with the risk of distant recurrence in patients receiving adjuvant trastuzumab.

HER2 can further potentiate immune evasion mechanisms by upregulating immune checkpoint proteins (i.e., PD-1, PD-L1, CTLA-4, and LAG3) to inhibit cytotoxic CD8+ T cell activity [164,165]. In gastric cancer, studies have reported a direct association between HER2 and PD-L1 expression, whereby patient-derived organoid models demonstrated higher PD-L1 levels in HER2+ tumors compared to HER2-negative counterparts. PD-L1 expression was shown to be reduced following HER2 pathway inhibition [166,167,168,169,170]. Chakrabati et al. [167] also observed that reduced PD-L1 expression in gastric cancer organoids correlated with a significant increase in cytotoxic T lymphocyte (CTL) proliferation and survival, supporting the role of HER2-induced PD-L1 in tumor immune evasion.

Previously, we described an inverse relationship between RKIP and PD-L1 expression via cross-talk signaling, via pathways such as MAPK and JAK/STAT, or cytokines such as IFN-γ and IL-1β [171] (Figure 2). These analyses were also corroborated by bioinformatic analyses, which showed a significant negative mRNA correlation between RKIP and PD-L1 across multiple cancer types, including Breast Invasive Carcinoma (BRCA) [171]. As such, one mechanism contributing to immune evasion could be the downregulation of RKIP to promote PD-L1 expression, considering HER2′s positive relationship with PD-L1 in the TME.

While much attention has been given to TILs as favorable prognostic markers in HER2+ BC, emerging evidence highlights the significance of tumor-associated macrophages (TAMs) in their density, localization, and polarization state in influencing the efficacy of HER2-targeted therapies [172] (Figure 2). Studies have shown that high TAM infiltration, especially of the M2-like (CD163+) subtype, is associated with poor disease-free survival in BC, including the HER2+ subset [173,174]. In HER2-overexpressing tumors, TAMs can directly affect the response to trastuzumab and pertuzumab [173,174,175]. TAMs also contribute to a pro-tumorigenic microenvironment by secreting cytokines such as interleukin-8 (IL-8) and CCL2, which promote cancer cell migration, stemness, and EMT [176,177]. Conversely, RKIP expression has been shown to inhibit the recruitment of TAMs through suppression of the chemokine ligand 5 (CCL5) in TNBC, thereby reducing tumor vascularization and metastatic potential [10,178,179]. Experimental restoration of RKIP expression in BC cells with low baseline RKIP levels significantly decreases TAM infiltration in vivo, as demonstrated in orthotopic mouse models [178]. TAMs isolated from RKIP-expressing tumors show reduced capacity to secrete metastasis-promoting factors like progranulin (PRGN) and tumor necrosis factor receptor 2 (TNFR2) [177]. These effects are mediated through RKIP’s inhibition of CCL5, which disrupts macrophage chemotaxis and alters the tumor microenvironment’s pro-metastatic signaling landscape. These findings show promising evidence for RKIP as a negative regulator of TAMs in the HER2 tumor landscape—capable of limiting the recruitment of immunosuppressive immune cells.

In the context of tumor plasticity and stemness, HER2+ tumors often show features of EMT, which is linked to an immune-evasive phenotype. Gupta and Srivastava have noted specific mechanisms whereby HER2 can regulate the TGFβ/SMAD pathway by inducing TGFβ de novo to promote EMT in BC [141]. In HER2+ patients with PTEN loss, continual administration of trastuzumab has also led to EMT induction and subtype switching towards TNBC [180]. Conversely, RKIP normally suppresses EMT through the inhibition of EMT-related gene products like SNAI1, TWIST, ZEB, Vimentin, and Notch1 while upregulating the epithelial E-Cadherin [181,182]. In addition, RKIP can inhibit the NF-κB/SNAIL/YY1 circuit, a pathway critical for the early steps of EMT induction [135,183]. Further, HER2 also positively upregulates EMT factors downregulated by RKIP, such as ZEB1 and TWIST1, to support the acquisition of stem-like properties [141,184,185]. TWIST1 has also been implicated in CD8+ T cell exhaustion through PD-L1 upregulation in BC cells [186]. Cross-talk between RKIP and HER2 highlights their roles in maintaining epithelial identity and immune visibility, especially in the context of HER2+ BC. Thus, restoring RKIP function may be a critical node for overcoming resistance in aggressive BC subtypes that are resistant to first-line therapies and for inhibiting immune evasion.

Previous studies have that shown cell lines with high RKIP (MCF-7) have low metastatic potential, and overexpressing RKIP in TNBC cell lines (MDA-MB-231) was able to reduce their migratory pattern [15]. In addition, a study by Kalpana et al. has shown RKIP can suppress BC cell invasion (specifically in triple-negative basal epithelial-like BC cell lines) through RhoA-mediated regulation of E-cadherin with EMT-associated proteins and transcription factors (e.g., Snail, Slug, and ZEB1/2) [187]. Many other studies have also established RKIP as a potential inhibitor of EMT in BC [178,188].

At the same time, HER2+-specific evidence directly demonstrating that RKIP loss drives EMT in patient-derived HER2+ samples or dedicated HER2+ cell-line models remains limited. Briefly, existing datasets have not stratified EMT phenotypes by both HER2 status and RKIP expression, and functional studies in HER2-amplified contexts are largely lacking. To avoid overstating the current clinical evidence, (1) RKIP robustly modulates EMT and invasion in BC models in general, and (2) by analogy and pathway overlap, RKIP loss is strongly suspected—but not yet conclusively proven—to promote EMT in HER2+ BC. Collectively, these findings reinforce the multifaceted role of RKIP–HER2 cross-talk in shaping an immunosuppressive TME (Table 4).

## 6. Bioinformatics Analyses

To explore the potential roles of HER2 and RKIP in cancer biology, we performed a series of bioinformatics analyses using publicly available data from The Cancer Genome Atlas (TCGA) [189] and the Clinical Proteomic Tumor Analysis Consortium (CPTAC) [190]. Our study focused on the co-expression, dysregulation, phosphorylation, and survival impact of these two molecules across various cancer types.

### 6.1. Expression and Correlation Analyses

**Figure 3:**
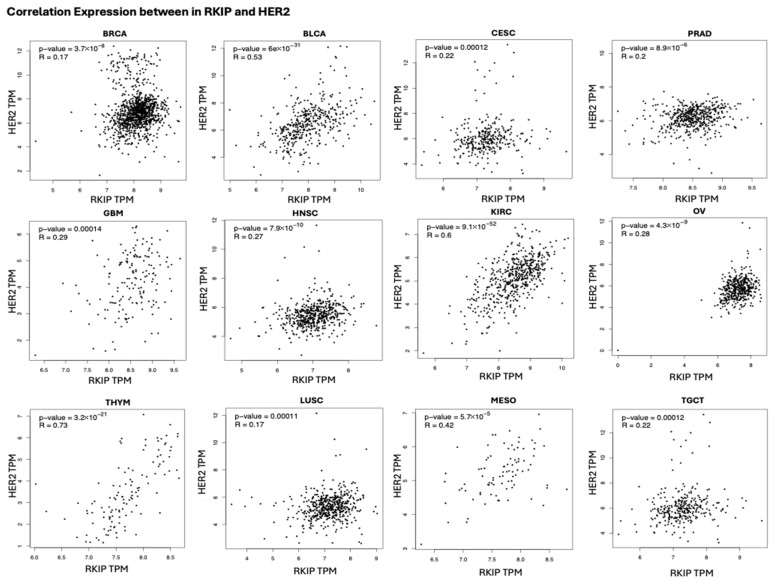
Significant positive correlations between RKIP and HER2 expressions in various cancers. Spearman correlation (R) and *p*-values are indicated in each graph. Graphs are produced using GEIPA with data from TCGA (Accessed on October 2024). Abbreviations: BRCA, Breast Invasive Carcinoma; BLCA, Bladder Urothelial Carcinoma; CESC, cervical squamous cell carcinoma and endocervical adenocarcinoma; PRAD, prostate adenocarcinoma; GBM, glioblastoma; HNSC, head and neck squamous cell carcinoma; KIRC, kidney renal clear cell carcinoma; OV, ovarian serous cystadenocarcinoma; THYM, thymoma; LUSC, lung squamous cell carcinoma; MESO, mesothelioma; TGCT, Testicular Germ Cell Tumors.

Utilizing the GEPIA2 platform with TCGA and GTEx datasets, we observed a significant positive correlation in mRNA expressions across 12 various cancers, including breast, bladder, glioblastoma, kidney, prostate cancers, etc. (Figure 3). This positive correlation was unexpected, given RKIP’s known role as a tumor suppressor. However, several factors could explain this observation. Firstly, mRNA expression does not always directly correlate with protein abundance, which aligns with our subsequent finding (discussed below) of inverse trends at the protein level in HER2+ BC. Secondly, mRNA analysis does not distinguish between the inactive phosphorylated and the active unphosphorylated forms of RKIP, and phosphorylated RKIP has been documented to have oncogenic roles in certain contexts.

In our study, mRNA-level analyses using large pan-cancer datasets showed an unexpected positive correlation of RKIP (PEBP1) expression across multiple tumor types, despite RKIP’s established function as a metastasis suppressor in experimental models. These transcriptomic data do not distinguish between active (unphosphorylated) and inactive (phosphorylated) RKIP, and, importantly, mRNA abundance frequently diverges from total and phospho-protein levels in clinical samples. Because currently available clinical datasets do not yet allow a clear separation of prognostic or predictive value for pRKIP versus unphosphorylated RKIP, these phospho-proteomic findings are hypothesis-generating, and the clinical significance of pRKIP and active RKIP remains unresolved.

### 6.2. Dysregulation of HER2 and RKIP Expressions in BC

In contrast to mRNA expression, protein-level analysis of RKIP using UALCAN [191] demonstrated a negative relationship pattern in BC subtypes, with RKIP significantly downregulated in HER2+, luminal, and triple-negative BC compared to normal tissues (Supplemental Figure S1). This downregulation is consistent with the previous literature and our hypothesis of an inverse relationship between RKIP and HER2 in the TME. To further elucidate the role of RKIP in cancer progression, we extended our investigation to analyze RKIP and pRKIP S60 protein expression patterns across various clinical stages and pan-cancer subtypes. This could provide useful insights to identify any subtype-specific trends for treatment stratification. Across the four clinical stages of BC, both RKIP and RKIP (S60) exhibit a similar decline in expression as BC progresses, with a few key differences between the two markers. The expression level of RKIP in normal tissue is notably higher compared to RKIP (S60), suggesting that RKIP has a higher baseline expression in normal tissues than its phosphorylated form. Additionally, RKIP (S60) exhibits a wider range of expression in the cancer stages, indicating greater variability in its levels during cancer progression. These differences between phosphorylated versions of RKIP are also highlighted in the pan-cancer proteomic profile of RKIP (Supplemental Figure S1).

Phosphorylated RKIP S60 is not currently recognized in the literature as a major regulatory site; in contrast, phosphorylation at Serine 153 (pRKIP-S153) is the best-characterized and functionally critical modification. Phosphorylation of S153 by PKC induces a conformational and binding shift in RKIP that leads to dissociation from Raf-1, thereby lifting its inhibitory effect on the MAP kinase pathway. This same modification promotes RKIP binding to GRK2, effectively switching its role from inhibiting Raf-1 to inhibiting G protein-coupled receptor (GPCR) signaling, with important consequences for cell proliferation and cancer metastasis. In our dataset, the “RKIP S60” signal originates from the UALCAN portal, where it represents a phosphosite-specific feature derived from large-scale phosphoproteomic analyses, in which any recurrently detected phosphoserine on RKIP is annotated as an individual variable. Thus, the S60-associated changes we report reflect a mass-spectrometry–defined phosphopeptide whose expression levels correlate with BC progression.

The pan-cancer subtypes K1 through K10 represent molecular classifications identified using mass-spectrometry-based proteomic data from CPTAC cohorts. These subtypes are characterized by distinct molecular signatures explained in these pan-cancer studies [192,193,194,195]. Briefly, K1 is associated with the proteasome complex and metabolic pathway proteins; K2 and K3 are linked to adaptive and innate immune responses, respectively; K4 represents basal-like BC with YAP1 and MYC target overexpression; K5 exhibits epithelial and normoxia signatures with oxidative phosphorylation proteins; K6 and K7 are stromal-related with distinct matrix protein expressions; K8 is associated with the Ras pathway; K9 is characterized by hemoglobin complex association; and K10 is linked to endoplasmic reticulum and steroid biosynthesis pathways. Supplemental Figure S1, which examines RKIP and RKIP (S60) expressions across these subtypes, reveals several important insights. Both forms show the highest RKIP expression in normal tissue, with significant downregulation in most cancer subtypes, supporting RKIP’s tumor suppressor role. Notably, RKIP shows no significant difference between normal tissue and the K1 subtype, while RKIP (S60) shows no significant difference between normal tissue and both K1 and K3 subtypes (Supplemental Figure S1). All other subtypes display significantly lower expression compared to normal tissue for both RKIP and RKIP (S60). The variability in expression across subtypes suggests subtype-specific regulation mechanisms. RKIP expression is relatively higher in K5 and K7 subtypes, while K2 and K6 exhibit the lowest levels. For RKIP (S60), K3 and K7 show relatively higher median expression levels. The greater variability observed in RKIP (S60) expression indicates more dynamic phosphorylation regulation in cancer. These expression patterns could have prognostic implications for targeting subtypes with lower RKIP expression for HER2+ BC.

Next, we looked at dysregulated expression levels of RKIP and HER2 between normal and tumor tissues. Consistent with the literature, HER2 was significantly overexpressed in BC tissues compared to normal tissues (*p* < 0.001) (Supplemental Figure S2). HER2 was also overexpressed in 12 out of the 33 TCGA cancer types (*p* < 0.01) (Supplemental Figure S3). Specifically, the 12 cancers include ACC, BRCA, CHOL, DLBC, GBM, KIRC, LUAD, PAAD, READ, SKCM, STAD, and THYM. In contrast, RKIP expression showed variability, with higher expression levels detected in THYM, CHOL, and DLBC. However, in tumors such as KIRC, PCPG, and SARC, downregulated RKIP expression levels were observed. In BRCA, RKIP is slightly upregulated; however, this is not statistically significant. Thus, the observed expression levels might reflect a subset of tumors where RKIP has not been entirely downregulated or where alternative regulatory mechanisms are in place.

### 6.3. Phosphorylation and Functional Profiling

Using UALCAN, we examined phosphorylation levels of HER2 and RKIP in BC. We observed significant decreases in phosphorylation at multiple HER2 amino acid sites (e.g., S1043, S1036, and Y1218) in breast tumor samples compared to normal tissues (Supplemental Figure S3), while HER2 phosphorylation at site Y1109 showed a significant increase in expression. We also observed significant phosphorylation of RKIP at site S60, but less than in normal tissues (Supplemental Figure S3), which might contribute to the regulatory interactions between RKIP and HER2. This finding corroborated our postulated positive correlation between HER2 and RKIP in BC (see Section 6.1).

### 6.4. Survival Analysis

Kaplan–Meier survival analyses and heatmaps were generated using GEIPA2 to assess the prognostic impact of HER2 and RKIP expressions on overall survival (OS) and disease-free survival (DFS) across various cancers. Each heatmap represents the log10-transformed hazard ratio (HR) values for a range of cancer types, where red indicates a positive HR (higher risk associated with higher expression) and blue indicates a negative HR (lower risk associated with higher expression). High HER2 expression acts as a significant adverse prognostic factor in Low-Grade Glioma (LGG), Ovarian Cancer (OV), and Skin Cutaneous Melanoma (SKCM), correlating with worse OS. Conversely, HER2 expression is a protective factor in various cancers such as kidney renal clear cell carcinoma (KIRC), MESO, COAD, and READ, where higher expression is linked to improved survival outcomes (Supplemental Figure S4). Similarly, RKIP expression is significantly associated with worse OS in SKCM. However, in many cancers, RKIP expression serves as a significant protective factor (LUAD, PAAD, UCEC, CESC, KIRC, LIHC, THCA), significantly improving both OS and DFS. The Kaplan–Meier plots further highlight the survival impact of RKIP and HER2 mRNA expression in BC. For RKIP, patients with low expression (red curve) have a better OS compared to those with high expression (blue curve), as shown by the log-rank *p*-value of 0.71 and HR suggesting worse survival outcomes for the high RKIP group. The possibilities of this were discussed earlier, pertaining to the roles between pRKIP and unphosphorylated RKIP. Similarly, for HER2, patients with high expression (red curve) show worse OS compared to the low HER2 group (blue curve), supported by a log-rank *p*-value of 0.09 and a corresponding HR (Supplemental Figure S4). These trends are suggestive and not statistically reliable. While the *p*-value meets conventional thresholds, we agree this reflects a modest trend rather than a robust prognostic signal, especially in TCGA-derived data prone to cohort heterogeneity.

Regarding the expectation that a classic tumor suppressor should show a strong association between low expression and worse overall survival, it is important to emphasize that RKIP functions primarily as a metastasis suppressor rather than a classic tumor suppressor like p53, meaning it permits primary tumor growth but inhibits later progression steps. This could explain the lack of clear OS prognostic significance in bulk analyses like TCGA, where high-RKIP tumors may still progress locally. In addition, associations with OS often lack statistical significance in large cohorts due to confounders like heterogeneous treatments, comorbidities, and competing risks (e.g., early deaths from non-metastatic causes).

Hagan et al.’s (CCR 2005) findings can also explain the positive mRNA correlations observed in bulk pan-cancer analyses like TCGA/GEPIA2, complementing the prior points on RKIP’s metastasis-suppressor role (rather than classic tumor suppression) and the disconnect between mRNA/protein forms [196].

The potential causes of the mRNA-protein discrepancy for RKIP include (1) post-transcriptional regulation (e.g., miRNA-mediated degradation, alternative splicing); (2) differential protein stability and turnover influenced by phosphorylation status or ubiquitination; (3) technical factors such as mRNA normalization in bulk TCGA data versus proteomics sensitivity for low-abundance proteins; and (4) intratumoral heterogeneity, where bulk mRNA averages mask protein-level losses in metastatic subclones.

## 7. Targeting the RKIP-HER2 Axis in HER2+ BC

Currently, several therapeutic modalities have been used to treat HER2 BC. Table 5 summarizes these modalities.

Multiple clinical studies demonstrate reduced or lost RKIP expression in lymph node metastases compared to matched primary breast tumors [10]. Additional evidence indicates that RKIP loss is associated with aggressive disease features and resistance to therapy [199]. However, HER2-specific longitudinal datasets remain limited. Various treatments for HER2-related BCs persist. Trastuzumab, lapatinib, pertuzumab, and margetuximab are standard therapies that have proven effective throughout years of use. Advances in understanding tumor immune surveillance have underscored the potential of immunotherapy as a treatment strategy for BC [148]. In this context, targeting the RKIP-HER2 axis and implementing strategies to restore RKIP function could present a novelty in therapeutic research as a cancer treatment, given RKIPs’ ability to block signaling pathways.

RKIP induction can occur in various ways. Some involve inhibiting RKIP repressors and suppressors such as SNAIL, BACH1, and EZH2 [39,63]. As EZH2 methyltransferase inhibitors, PCR2 inhibitors, and degradation take action, the induction of RKIP expression can be achieved as EZH2 is directly involved in suppressing RKIP and regulating its transcription in BC [39,63]. In particular, Ren et al. [39] demonstrated that RKIP inhibition leads to cancer cell invasion by EZH2. This suggests that by inhibiting EZH2, RKIP expression may be induced, which may impede tumor proliferation and progression. Other EZH2 inhibitors that may induce RKIP and inhibit HER2 include Tazemetostat (EPZ-6438, also known as Tazverik), GSK126, EPZ005687, and SHR2554. Tazemetostat can incorporate oncogenic signaling and immune modulation in inducing RKIP and inhibiting HER2 for cancer treatment. It has undergone several preclinical and clinical trials, demonstrating efficacy and high anti-cancer activity [200]. GSK126 is another selective EZH2 inhibitor that demonstrates anti-cancer properties by reducing H3K27me3 levels in tumors and altering oncogenic pathways. GSK126 has 1000 times more selectivity for EZH2 than the other 20 methyltransferases [200]. On the other hand, EPZ005687 was the first EZH2-specific inhibitor with high EZH2 affinity and has over 500-fold selectivity. However, its unsatisfactory pharmacokinetic characteristics result in limited clinical application [200]. On the same note, SHR2554 is a small-molecule EZH2 inhibitor and a growing therapeutic agent for cancer [200].

Furthermore, NO mediation has been shown to be effective in the chemical induction of RKIP [37,191,201,202]. An NO donor, DETANONOate, provides NO, and the overexpression of RKIP mimics NO in tumor cells, sensitizing them to apoptosis. As NO induces RKIP, SNAIL is repressed downstream of NFκB [36]. This NO donor also inhibits SNAIL, YY1, and NFκB, disrupting the NFκB/SNAIL/YY1/RKIP feedback loop [36,190,191]. NO nitrosylates YY1 and inhibits its DNA-binding activity; hence, it inhibits SNAIL expression, a repressor of RKIP, leading to RKIP expression [203].

Similarly, RKIP can be induced by inhibiting the NF-κB and SNAIL pathways. Proteasome inhibitors such as NPI-0052 or NF-κB inhibitors like Dehydroxymethylepoxyquinomicin (DHMEQ) [142] have shown effectiveness. NPI-0052, for example, blocks the transcription and expression of NF-κB promoter activity, leading to RKIP induction [36,204]. Baritaki et al. [36] also demonstrated that treatment with DHMEQ reduced SNAIL mRNA and increased RKIP mRNA levels. Findings also showed that treatment with DHMEQ enhanced apoptosis when combined with CDDP or TRAIL. With that said, both NPI-0052 and DHMEQ sensitize tumors to apoptosis by suppressing NF-κB and anti-apoptotic targets like Bcl-xL [36]. SNAIL siRNA treatment is another agent used to induce RKIP expression [142]. Immunomodulatory drugs, including antibodies like rituximab and LFB-R603, can also upregulate RKIP by targeting pathways such as NF-κB, PI3K/Akt, and ERK1/2 [142,205].

Another innovative approach that has emerged as a promising therapeutic for BC and restoring RKIP function involves Proteolysis-Targeting Chimeras (PROTACs) [206]. By harnessing a cell’s ubiquitin-proteasome system, PROTACs allocate targeted protein degradation and help overcome HER2 therapy resistance [206]. Specifically, in a study by Zhang et al. [206], PI3K PROTAC was examined for its ability to overcome HER2-targeted therapy resistance through induced PIK3CA gene mutation. Results revealed that the PI3K PROTAC demonstrated excellent efficacy in HER2+ resistant cell lines. Further analysis determined whether PI3K PROTAC had the potential to enhance the therapeutic effects of lapatinib, showing that PI3K PROTAC increased the sensitivity of lapatinib in BT474 and HER2+ cell lines [206]. Although drug resistance to anti-HER2 medications may develop due to PI3K-Akt-mTOR activation, PI3K inhibitors have the potential to reverse such resistance and resensitize cell lines to undergo repeated cycles of protein degradation, a mechanism that is reminiscent of RKIP’s inhibitory role on HER2-driven signaling through the same pathway [206]. PI3K PROTAC has demonstrated efficacy through degrading p110α and p85β [206]. However, other PROTACs, such as EZH2 PROTACs, can suppress BC cell growth by degrading EZH2. This degradation leads to lower target gene expression, contributing to the suppression of cancer cell growth [207]. Off-target effects, such as the degradation of non-specific proteins, variability in tumor microenvironment, and immunogenicity, are factors that may hinder and pose challenges to PROTAC effectiveness, similar to other therapeutic approaches.

In addition to the promise of PROTACs in overcoming HER2 therapy resistance, nanotechnology has advanced the development of novel therapeutic approaches for treating BC. A study by Zhang et al. [208] demonstrated the design of non-toxic transformable peptides that bind to HER2 on cancer cells. These peptides then transform into nanofibrils upon binding, disrupting HER2 dimerization and signaling, ultimately leading to cancer cell death. This process blocks cell proliferation, impeding signaling pathways supporting tumor progression [208].

Photodynamic therapy (PDT) also emerges as an innovative therapeutic approach to induce RKIP and reverse resistance [209]. PDT combines photosensitizers, light, and oxygen to produce reactive oxygen species (ROS) and NO, which can promote cell apoptosis and autophagy [209,210,211]. PDT enhances its inhibitory effects through these mechanisms, inducing RKIP expression and disrupting resistance pathways.

Further supporting innovative strategies, a study by Yun et al. [40] identified RKIP as a prominent BC biomarker. The study found that RKIP induces the expression of let-7, suppressing tumor progression by blocking novel targets of let-7, including BACH1 and HMGA2. By blocking these targets, RKIP contributes to BC development and suppression, thereby reinforcing the potential RKIP holds as a novel form of treatment in combating HER2+ BC.

The potential of immune checkpoint inhibitors (ICIs) has also emerged in many clinical trials to overcome immune evasion mechanisms in HER2+ BC. For example, the PANACEA trial assessed a PD-1 inhibitor with trastuzumab in trastuzumab-resistant, advanced HER2+ BC through two-phase cohorts, which demonstrated efficacy and safety in treating patients with programmed cell death 1 ligand 1 (PD-L1)-positive tumors that are also trastuzumab-resistant in advanced HER2+ BC [212]. However, the KATE2 trial, a randomized, double-blind, placebo-controlled study, evaluated the combination of atezolizumab, a PD-L1 inhibitor, with trastuzumab emtansine in patients with HER2+ advanced BC [213]. Results showed that the combination did not provide a meaningful improvement, as the progression-free survival median was 8.2 months for the atezolizumab group and 6.8 months for the placebo group, with a 0.82 hazard ratio (*p* = 0.33), highlighting no meaningful clinical benefit [213].

Despite advancements in HER2-targeted therapies, resistance to these treatments remains a major challenge, and primary resistance can occur due to impaired drug binding or constitutive activation of downstream signaling pathways, while acquired resistance often arises from the selection of subclones with genetic alterations that bypass HER2 inhibition [214]. Mechanisms such as activation of parallel pathways (e.g., MET and FGFR), mutations in PI3K/Akt, and metabolic reprogramming contribute to therapeutic resistance [215]. Additionally, HER2 overexpression itself can play a role in resistance by promoting receptor dimerization and sustained signaling, even in the presence of targeted agents, which can potentially lead to PI3K/Akt activation, contributing to treatment resistance [216]. Moreover, reduced immune system activation in the tumor microenvironment limits the effectiveness of immunotherapies.

ADCs represent a targeted therapeutic strategy that combines the specificity of monoclonal antibodies with the cytotoxic potency of small-molecule payloads [217]. In HER2+ BC, trastuzumab deruxtecan has demonstrated superior clinical efficacy compared to earlier HER2-directed therapies, owing to its high drug-to-antibody ratio, cleavable linker, and membrane-permeable payload, which enable killing in heterogeneous tumors [218,219].

Beyond direct cytotoxicity, emerging evidence indicates that ADCs can exert immunomodulatory effects within the TME [217,220]. ADC-induced tumor cell death can promote immunogenic cell death, leading to enhanced antigen release, dendritic cell activation, and improved CD8+ T-cell priming [220]. In addition, HER2-targeted ADCs retain Fc-mediated immune functions, including antibody-dependent cellular cytotoxicity (ADCC), further contributing to anti-tumor immune responses [221,222,223].

Given the central role of immune evasion mechanisms in therapy resistance, ADCs represent a clinically relevant platform that intersects targeted cytotoxicity with immune modulation.

## 8. Discussion, Challenges, and Future Perspectives

While RKIP’s role as a tumor suppressor has been extensively studied over the past decades, its function in regulating immune responses has only recently begun to be elucidated in vivo. Emerging evidence highlights RKIP as a promising immune modulator in BC, a finding of relevance given the persistent challenge of immune evasion in HER2+ BC, especially in patients with de novo stage IV disease or acquired resistance to trastuzumab. We suspect an inverse relationship between RKIP and HER2 that is central to immune suppression and evasion. The downregulation of RKIP correlates with overexpression of HER2, contributing to an immunosuppressive TME and enhanced tumor immune evasion. These findings provide compelling evidence for the existence of a dysregulated RKIP-HER2 axis as a critical regulator of anti-tumor immunity in HER2+ BC.

RKIP has long been established as a metastasis suppressor and negative regulator of the MAPK/ERK signaling pathway, and its deficiency correlates with HER2 activation. HER2 signaling is linked to immune evasion through downstream activation of the PI3K/Akt and STAT3 pathways, upregulating immune checkpoint molecules such as PD-L1 and promoting the secretion of immunosuppressive cytokines. These changes not only impair cytotoxic T-cell infiltration but also enhance the recruitment and polarization of regulatory T cells and myeloid-derived suppressor cells, creating a permissive niche for tumor progression. Restoration of RKIP expression in HER2-overexpressing cells results in diminished PD-L1 expression and improves T-cell activation, underscoring the therapeutic potential of modulating this axis.

Recent reviews and original studies have highlighted how dysregulated oncogenic signaling pathways contribute to immune escape and resistance to both targeted therapies and immunotherapy in BC [147,224]. Additional recent reports have emphasized the role of HER2-directed therapies, including ADCs, in reshaping the TME and improving anti-tumor immunity, particularly when combined with immune-based strategies [222,223,224,225].

Compelling data additionally support the cross-talk between HER2 and RKIP within the tumor milieu. Beyond its direct effects on tumor cells, RKIP modulates the TME by restricting TAM recruitment through the suppression of chemokines, such as CCL5, and by blocking HMGA2-mediated pathways [178]. Since TAMs are major sources of PD-L1 and contribute to resistance to HER2-targeted therapies, RKIP’s ability to limit TAM infiltration may enhance anti-tumor immunity and improve therapeutic efficacy.

Our bioinformatics analyses revealed an inverse correlation between RKIP expression and HER2+ BC across multiple subtypes. Interestingly, mRNA-level analyses showed a positive association between RKIP and HER2, potentially reflecting the prevalence of phosphorylated (and thus functionally inactive) RKIP isoforms. To address this discrepancy, we have evaluated multiple strategies—both direct transcriptional activation and indirect pathway modulation—to upregulate functional RKIP in HER2+ BC models, emphasizing the need for systematic validation through preclinical investigations followed by clinical translation. We also found that RKIP-low/HER2-high tumors were associated with poor infiltration of CD8+ T cells and higher expression of immune exhaustion markers. These findings align with recent observations that HER2-positive tumors with low immunogenicity exhibit limited response to immune checkpoint blockade (ICB).

Recent system-level analyses further reveal that RKIP and the pro-metastatic transcription factor BACH1 operate as mutually antagonistic regulators of cancer cell plasticity and immune evasion [226]. While BACH1 drives EMT, stemness, and immune checkpoint expression, RKIP maintains an epithelial phenotype, suppresses EMT, and correlates with improved patient survival. This antagonism extends to the regulation of immune cell infiltration and checkpoint protein expression, reinforcing RKIP’s broader impact in the TME. Although some resistance mechanisms, such as epigenetic alterations and the reactivation of the PI3K/Akt/mTOR axis, have been characterized in both preclinical and clinical settings, further research is needed to refine combination therapies to delay or overcome resistance in metastatic HER2+ BC [28].

Across various cancers, RKIP plays a pleiotropic role in suppressing metastasis and reshaping the TME. By downregulating checkpoints, inhibiting pro-metastatic signaling, and limiting the recruitment of immunosuppressive TAMs, RKIP emerges as a central node in the regulation of cancer-immune interactions. Strategies to restore or mimic RKIP function, or to combine HER2-targeted therapies with TAM or checkpoint inhibitors, offer a promising route to enhance efficacy and overcome resistance.

Clinically, the inverse relationship between RKIP and HER2 expression may have prognostic significance. Targeting the RKIP-HER2 axis could reprogram the TME and sensitize tumors to immunotherapy. Combinatorial strategies involving HER2 inhibitors (e.g., trastuzumab, pertuzumab) and immunomodulatory agents, particularly in RKIP-low tumors, are warranted. Additionally, pharmacologic or gene therapy-based restoration of RKIP may represent a novel strategy to enhance tumor immunogenicity.

Current evidence supports a reproducible inverse relationship between RKIP and HER2 signaling activity at the mechanistic and bioinformatic levels; however, definitive prognostic significance has not yet been established. While inverse expression patterns are observed across multiple datasets, survival correlations are modest and cohort-dependent. We therefore emphasize that the RKIP-HER2 relationship is biologically meaningful but requires validation in large, clinically annotated HER2+ BC cohorts before being considered prognostic.

## Figures and Tables

**Table 1:** RKIP expression across BC molecular subtypes with emphasis on HER2+ BC.

BC Subtype	RKIP Expression Level	Key Observations	References
Luminal A	High	RKIP expression is generally preserved at both the mRNA and protein levels in luminal A tumors compared to other subtypes.	[3,23]
Luminal B	Moderate to High	RKIP expression remains relatively higher than in non-luminal subtypes but may show some reduction compared to luminal A tumors.	[3,23]
HER2-enriched (HER2+ BC)	Low (protein level)	RKIP expression is frequently reduced in HER2+ tumors compared to normal breast tissue and luminal subtypes.	[17,22]
Basal-like	Low	Marked reduction in RKIP expression relative to luminal subtypes.	[22,23]
Claudin-low	Low	RKIP expression is diminished.	[22,23]

**Table 3:** Regulation of HER2 expression in HER2+ BC.

Types of Regulation	Factors Involved	References
Transcriptional	- HER2-p185neu nuclear complex - Transcription factors: TFAP2, Sp1, PBP, YY1, ETS, YB-1, EGR2 (upregulate HER2); MYB, FOXP3, GATA4, PEA3, MBP-1, NOTCH, RBP-Jk (downregulate HER2); E1A represses HER2 via p300/CBP inhibition	[67,68,69,70,71,72,73,74,75,76,78,79,81,82,83,84,85,86,87,88,89,94]
Epigenetic	- Histone modifications (e.g., H3K4me3, H3K9ac) - HER2 gene body enhancer (HGE) - DNA methylation/demethylation of the HER2 promoter - ncRNAs interfering with chromatin accessibility	[11,89,95,96,97,98,99,100,101,102,103]
Post-Transcriptional	- lncRNA: LINK-A - miR-342-5p - miR-124 and miR-193a-3p - miR-96, miR-10b, and miR-17 - miR-335-5p - miR-148a	[104,105,106,107,108,109,110,111,112,113,114,115,116,117,118,119]
Translational	- miRNAs targeting 3′UTR - Hsp90 - Proteasome inhibitors (PIs) - PTPN18	[11,108,120,121,122,123,124,125,126,127,128,129,130]

**Table 4:** Interplay between RKIP and HER2 signaling in HER2+ BC.

Model/Context	RKIP Status	HER2 Status	Mechanistic/Immune Effects	Phenotype Related to Immune Evasion or Progression	Key Finding	References
HER2-enriched subtype	Low	High	Loss of RKIP permits sustained MAPK, NF-κB, and PI3K/Akt signaling downstream of HER2	Immunosuppressive TME, increased tumor aggressiveness	Inverse correlation between RKIP and HER2 expression	[22]
HER2-enriched subtype	Low	High	HER2 signaling inversely regulates RKIP expression; dose-dependent reduction in RKIP upon HER2 modulation	Associated with increased invasiveness and aggressive HER2+ phenotype	RKIP is negatively correlated with HER2 status and serves as a negative predictor of HER2; HER2-enriched tumors show among the lowest RKIP levels	[23,26]
HER2+ BC with EMT features	Low	High	HER2 induces EMT via SNAIL, ZEB1, STAT3; RKIP inhibits NF-κB/SNAIL/YY1 circuit	EMT-associated immune evasion and metastatic progression	RKIP loss facilitates EMT, whereby EMT is a key feature of immune escape in HER2+ BC	[134,141,142,143]
HER2+ BC tumor microenvironment	Low	High	HER2 upregulates PD-L1 via MAPK and PI3K/Akt; RKIP inversely correlates with PD-L1 expression	CD8+ T-cell inhibition, checkpoint-mediated immune evasion	RKIP downregulation contributes to PD-L1–mediated immune suppression	[164,165,166,167,168,169,170,171]
HER2+ BC with TAM infiltration	Low	High	HER2 promotes M2-like TAM recruitment; RKIP suppresses CCL5-mediated macrophage chemotaxis	Immunosuppressive, pro-tumorigenic macrophage-rich TME	RKIP expression limits TAM infiltration and metastatic signaling	[172,173,174,175,176,177,178,179]

**Table 5:** HER2-targeted therapies for BC overview.

HER2-Targeted Therapy	Approval Year	Mechanism of Action	Use	References
Trastuzumab (Herceptin^®^, Genentech, Inc., San Francisco, CA, USA)	1998	Binds to HER2′s extracellular domain, subsequently inhibiting the PI3K and MAPK pathways, suppressing cellular growth	First targeted molecular treatment for HER2+ BC	[197]
Lapatinib (Tykerb^®^, GlaxoSmithKline (GSK), London, UK)	2007	HER1/HER2 kinase inhibitor	Frequently combined with capecitabine to treat metastatic BC	[197]
Pertuzumab (Perjeta^®^, Genentech, Inc., San Francisco, CA, USA)	2012	HER2/HER3 antibody that binds to HER2′s extracellular dimerization domain II	Used with trastuzumab and docetaxel for HER2-positive cases	[197]
Margetuximab (Margenza^®^, MacroGenics, Rockville, MD, USA)	2020	Monoclonal antibody (mAb) similar to trastuzumab, derived from 4D5	Can be paired with chemotherapy, effective for HER2-positive BC (SOPHIA trial)	[11,198]

## Data Availability

No new data were created or analyzed in this study.

## Supplementary Materials

The following supporting information can be downloaded at: https://www.mdpi.com/article/10.3390/cells15040319/s1, Figure S1: RKIP and RKIP (S60) Proteomic Expressions. RKIP and RKIP (S60) proteomic expression profiles across major subclasses (A), clinical stages (B), and pan-cancer subtypes (C) of breast cancer (BC), analyzed using UALCAN from CPTAC datasets. Boxplots represent the Z-scores of expression levels, with statistical significance denoted by asterisks (* *p* < 0.05, ** *p* < 0.01, *** *p* < 0.001, **** *p* < 0.0001, ns = not significant). Each panel highlights the differential expression trends of RKIP and RKIP (S60) in normal tissue and cancer categories; Figure S2: Expression Analysis of RKIP and HER2 Expression in Various Cancers. Box plots illustrate the differential expression of HER2 and RKIP in tumor versus normal tissues across various cancer types. The analysis highlights significant dysregulation (*p* < 0.01) of these genes in tumor samples compared to normal tissues. Gene expression data were retrieved from TCGA. Abbreviations: ACC, Adrenocortical carcinoma; CHOL, Cholangiocarcinoma; DLBC, Lymphoid Neoplasm Diffuse Large B-cell Lymphoma; READ, Rectum adenocarcinoma; STAD, Stomach adenocarcinoma; Figure S3: RKIP and HER2 Phosphorylation Sites. The figure shows box plots of phosphorylation levels of HER2 at different phosphorylation sites (T671, S1036, S1043, Y1109, and Y1218) and RKIP at S60. Phosphorylation levels are represented by Z-values, indicating deviations from the median. Differences between normal tissues (*n* = 18) and primary tumors (*n* = 125) are shown, with *p*-values indicating statistical significance for each phosphorylation site. The data is derived from log2 spectral count ratio values from the CPTAC dataset, normalized within each sample and across samples to account for variations; Figure S4: Heatmaps and Kaplan-Meier plots of RKIP and HER2 mRNA expression in various cancers using GEPIA 2 with TCGA datasets. (A) Heatmaps show log10-transformed hazard ratios (HR) for overall survival (OS) and disease-free survival (DFS), with significant associations outlined in bold. Red indicates adverse prognosis (higher expression linked to worse survival), while blue indicates protective prognosis. (B) Kaplan-Meier plots demonstrate OS for RKIP and HER2 in BC, with significant differences between high- and low-expression groups.

## Abbreviations

The following abbreviations are used in this manuscript:

Akt	Protein Kinase B
BACH1	Basic Leucine Zipper Transcription Factor 1
BC	Breast Cancer
BRCA	Breast Invasive Carcinoma
CCL5	Chemokine Ligand 5
CD8+	Cluster of Differentiation 8 Positive
CHOL	Cholangiocarcinoma
CPTAC	Clinical Proteomic Tumor Analysis Consortium
DFS	Disease-Free Survival
DLBC	Diffuse Large B-Cell Lymphoma
E1A	Adenovirus Early Region 1A
EGR2	Early Growth Response Protein 2
EMT	Epithelial–Mesenchymal Transition
ERK	Extracellular Signal-Regulated Kinase
ETS	E26 Transformation-Specific
EZH2	Enhancer of Zeste Homolog 2
FOXP3	Forkhead Box Protein P3
GATA4	GATA-Binding Protein 4
GRK2	G-Coupled Receptor Kinase 2
HER2	Human Epidermal Growth Factor Receptor 2
HGE	HER2 Gene Body Enhancer
Hsp90	Heat Shock Protein 90
KIRC	Kidney Renal Clear Cell Carcinoma
lncRNA	Long Non-Coding RNA
MAPK	Mitogen-Activated Protein Kinase
MBP-1	c-Myc Promoter-Binding Protein-1
MDSCs	Myeloid-Derived Suppressor Cells
miRNA	microRNA
mTOR	Mammalian Target of Rapamycin
MYB	Myeloblastosis Oncogene
NF-κB	Nuclear Factor Kappa B
NOTCH	Notch Receptor
OS	Overall Survival
pCR	Pathologic Complete Response
PD-1	Programmed Death-1
PD-L1	Programmed Death-Ligand 1
PEA3	Polyomavirus Enhancer Activator 3
PI3K	Phosphoinositide 3-Kinase
PIs	Proteasome Inhibitors
PKCζ	Protein Kinase C Zeta
PRC2	Polycomb Repressive Complex 2
PRLR	Prolactin Receptor
RBP-Jk	Recombination Signal Binding Protein for Immunoglobulin Kappa J Region
READ	Rectum Adenocarcinoma
RKIP	Raf Kinase Inhibitor Protein
S60	Phosphorylation Sites
SKCM	Skin Cutaneous Melanoma
Sp1	Specificity Protein 1
STAT3	Signal Transducer and Activator of Transcription 3
TAMs	Tumor-Associated Macrophages
TCGA	The Cancer Genome Atlas
THYM	Thymoma
TFAP2	Transcription Factor Activator Protein 2
TFs	Transcription Factors
TNBC	Triple-Negative Breast Cancer
Tregs	Regulatory T Cells
UALCAN	University of Alabama at Birmingham Cancer Data Portal
YY1	Yin Yang 1
