# Supplementary material for: The Role of Raf Kinase Inhibitor Protein (RKIP) in HER2+ Breast Cancer Immune Evasion
Source: Cells. 2026 Feb 8;15(4):319. doi: 10.3390/cells15040319 (PMC12939971; doi:10.3390/cells15040319)
Supplement: Supplementary file 1 [file cells-15-00319-s001.zip › cells-4015536-supplementary.pdf]

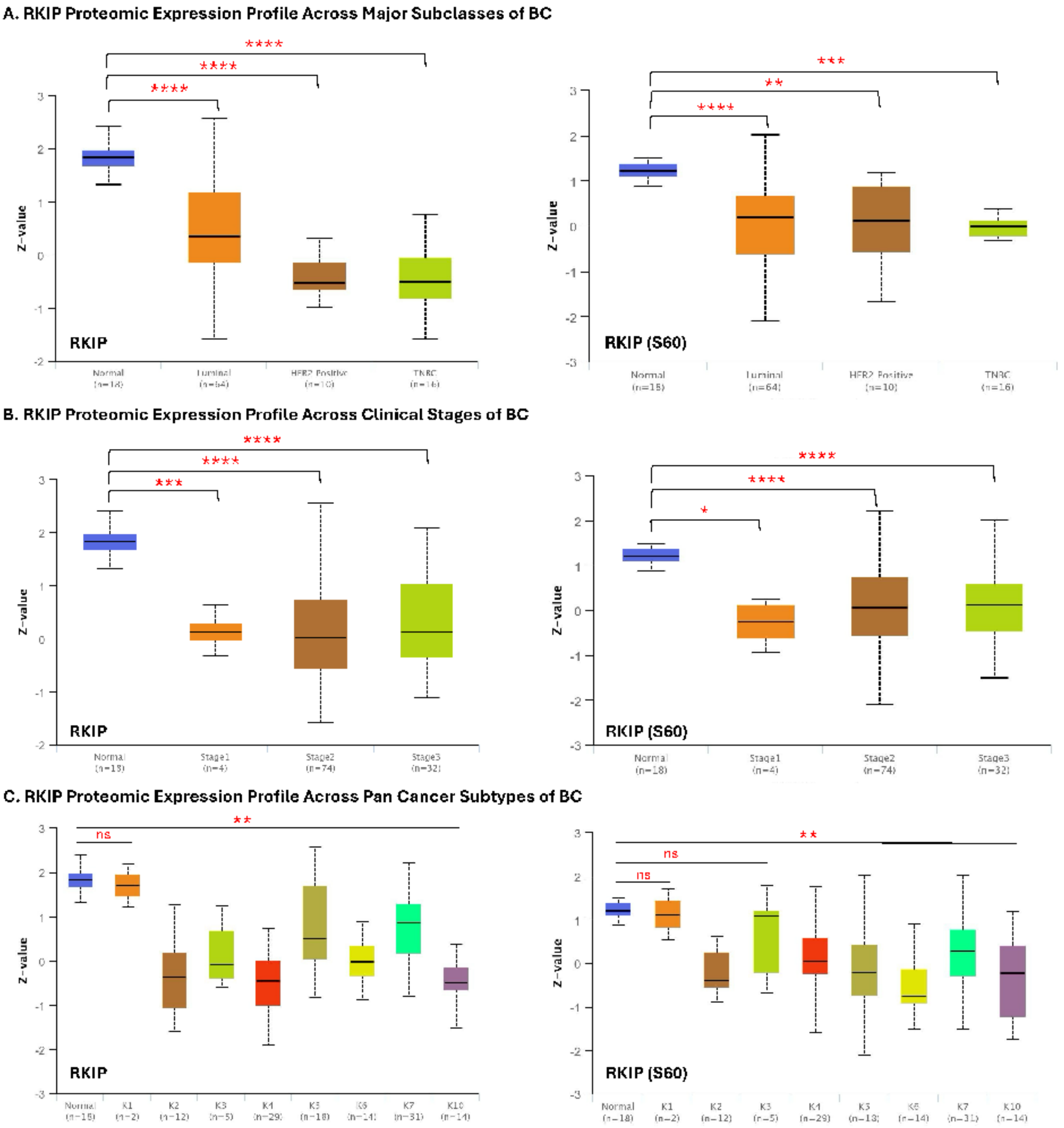

Supplemental Figure S1. RKIP and RKIP (S60) Proteomic Expressions. RKIP and RKIP (S60) proteomic expression profiles across major subclasses (A), clinical stages (B), and pan-cancer subtypes (C) of breast cancer (BC), analyzed using UALCAN from CPTAC datasets. Boxplots represent the Z-scores of expression levels, with statistical significance denoted by asterisks (* $p < 0.05$, ** $p < 0.01$, *** $p < 0.001$, **** $p < 0.0001$, ns = not significant). Each panel highlights the differential expression trends of RKIP and RKIP (S60) in normal tissue and cancer categories.

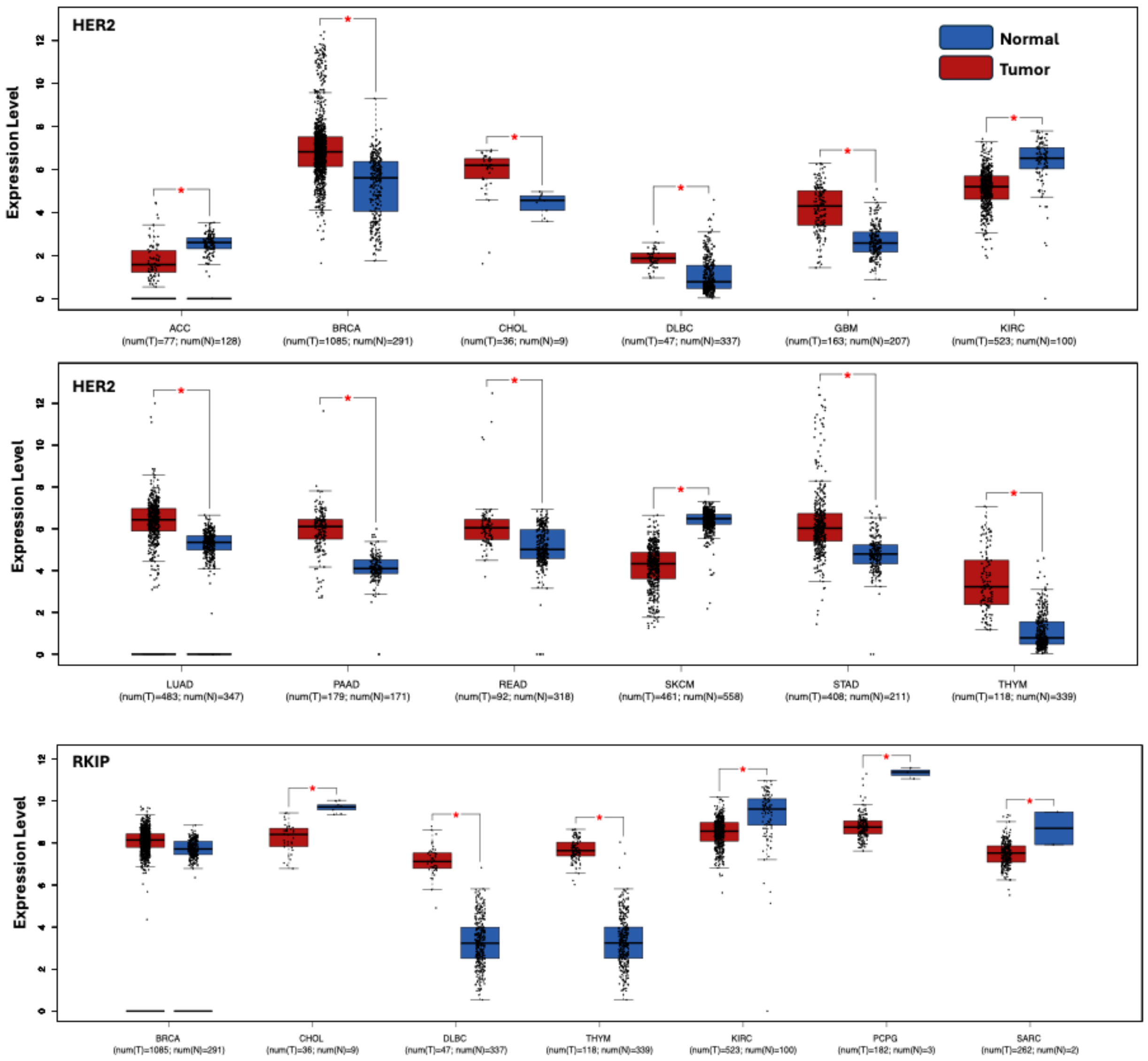

Supplemental Figure S2. Expression Analysis of RKIP and HER2 Expression in Various Cancers. Box plots illustrate the differential expression of HER2 and RKIP in tumor versus normal tissues across various cancer types. The analysis highlights significant dysregulation ($p < 0.01$) of these genes in tumor samples compared to normal tissues. Gene expression data were retrieved from TCGA. Abbreviations: ACC, Adrenocortical carcinoma; CHOL, Cholangiocarcinoma; DLBC, Lymphoid Neoplasm Diffuse Large B-cell Lymphoma; READ, Rectum adenocarcinoma; STAD, Stomach adenocarcinoma.

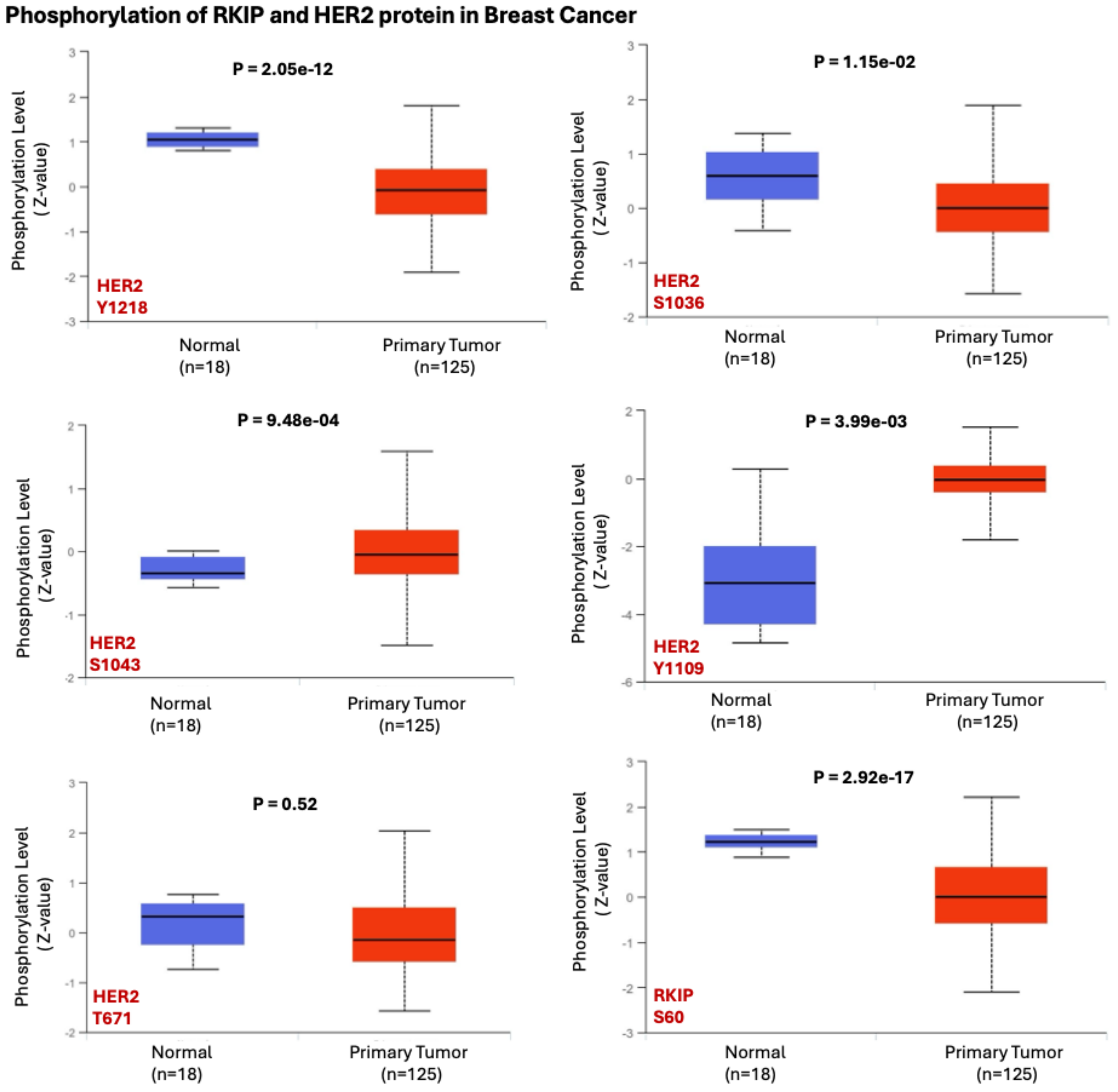

Supplemental Figure S3. RKIP and HER2 Phosphorylation Sites. The figure shows box plots of phosphorylation levels of HER2 at different phosphorylation sites (T671, S1036, S1043, Y1109, and Y1218) and RKIP at S60. Phosphorylation levels are represented by Z-values, indicating deviations from the median. Differences between normal tissues (n = 18) and primary tumors (n = 125) are shown, with p-values indicating statistical significance for each phosphorylation site. The data is derived from log2 spectral count ratio values from the CPTAC dataset, normalized within each sample and across samples to account for variations.

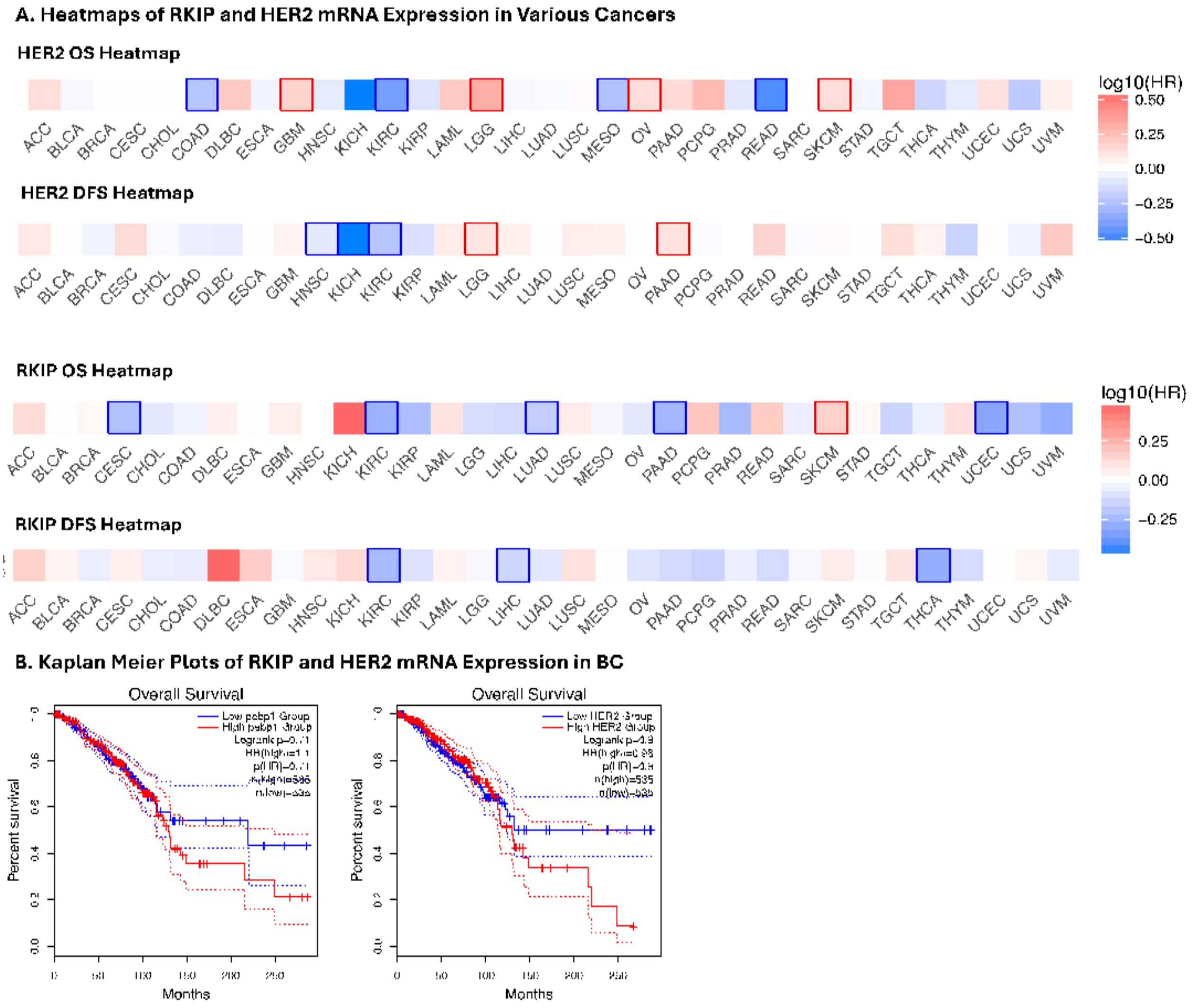

Supplemental Figure S4. Heatmaps and Kaplan-Meier plots of RKIP and HER2 mRNA expression in various cancers using GEPIA 2 with TCGA datasets. (A) Heatmaps show log10-transformed hazard ratios (HR) for overall survival (OS) and disease-free survival (DFS), with significant associations outlined in bold. Red indicates adverse prognosis (higher expression linked to worse survival), while blue indicates protective prognosis. (B) Kaplan-Meier plots demonstrate OS for RKIP and HER2 in BC, with significant differences between high- and low-expression groups.
